# ES-DPR: A DOA-Based Method for Passive Localization in Indoor Environments

**DOI:** 10.3390/s19112482

**Published:** 2019-05-30

**Authors:** Zhang Chen, Jinlong Wang

**Affiliations:** 1 College of Communications Engineering, PLA Army Engineering University, Nanjing 210007, China; wjl543@sina.com; 2 The Sixty-third Institute, National University of Defense Technology, Nanjing 210007, China

**Keywords:** indoor passive positioning, direction of arrival (DOA), ES-DOA, uniform linear antenna, coherent signal source, direct-path recognition

## Abstract

In this paper, we propose a novel indoor passive localization approach called eigenspace-based DOA with direct-path recognition (ES-DPR), based on a DOA estimation algorithm with multiple omnidirectional antennas deployed in a uniform linear array (ULA). To address the multipath propagation interference problem in the indoor environments, we utilize the azimuth and RSS estimation results, which are calculated by using the eigenspace-based DOA (ES-DOA) algorithm, in a novel style. A direct-path bearing recognition algorithm is introduced to identify the real DOA of the signal source in different indoor environments, by combining the azimuth and RSS estimation with ensemble learning methods. Numerical simulations are conducted to verify the validity and superiority of the proposed method. The results show that the proposed ES-DPR method can achieve high resolution and has strong anti-noise capability in dealing with the multipath signals, and the direct-path recognition algorithm is reliable and robust in different indoor environments, even in undetectable direct-path conditions.

## 1. Introduction

Recently, indoor location-based services (LBS) are in strong demand of many commercial and industrial applications, such as logistics, security, emergency and underground parking. Hence, indoor positioning systems (IPS) employing various technologies, such as wireless radio signals, infrared, visual surveillance, ultrasound or sound, inertial measurement units (IMU), and magnetic fields, have been developed to obtain accurate indoor location determinations, which is essential to LBS [1]. Due to the rapid development of mobile devices and wireless techniques, indoor localization with wireless signals has attracted considerable research efforts. In general, these localization approaches can be divided into several categories by the types of signal measurements, such as received signal strength (RSS), time of arrival (TOA), time difference of arrival (TDOA), and direction of arrival (DOA) [2].

RSS-based IPSs are very popular for their inherent simplicity and pervasive support by most wireless devices. In the last decade, a significant research effort has been directed towards indoor localization utilizing location fingerprinting techniques [3,4,5,6,7] that match the fingerprint of the RSS that is location dependent, such as RADAR [3] and Horus [4]. Although RSS-based localization methods are simple and easy to implement, there are certain shortcomings. The positioning performance is sometimes not robust or reliable, as the RSS measurements are vulnerable to variations in the indoor environment. Moreover, the survey and calibration process of fingerprints are rather time-consuming and labour intensive, which may render the fingerprinting techniques impractical for deployment over large areas [6].

The TOA approach calculates the distance between the target and the sensor by measuring the travelling time needed by the signal [8]. The target can be localized to a circle centred on the sensor with a radius estimated through the TOA; at least three sensors are required to identify the exact location. In [9], performance evaluations of several TOA/TDOA estimation algorithms employed a stochastic radio channel model conforming to wireless fidelity (WiFi) g/n/ac. Vasisht et al. [10] proposed a TOA algorithm that can locate with decimetre-level accuracy. In [11], a novel TOA-based positioning technique was proposed to obtain improved resolution under limited signal bandwidth without heavy calculation loading. Nevertheless, the nanosecond scale requirements of synchronized targets and sensors limit the widespread application of TOA-based IPS.

Instead of absolute time measurements, the TDOA method examines the relative time difference at which the signal arrives at different sensors that are clock synchronized. The target is supposed to be on a hyperboloid for each TDOA measurement with a constant difference between the two sensors. In [12], Exel et al. realized nanosecond accuracy by extracting timestamps in an IEEE 802.11b wireless local area network (WLAN) with a special synchronized receiver. In [13], an enhanced TDOA approach based on the RSS-assisted cross-correlation method was proposed to handle multipath interference in indoor environments. However, under situations in which TDOA measurements are strongly affected by multipath interference, it is not suitable for indoor localization scenarios.

DOA-based techniques include the calculation of the angle at the sensor of the signal arriving from the transmitter. Some DOA-based IPSs [14,15] can achieve sub-metre-level localization accuracy by incorporating antenna arrays and super-resolution algorithms. However, these DOA-based IPSs require a priori knowledge of the received signal and multiple base-station (BS) deployments.

### 1.1. Problem Statement

Most of the previously mentioned IPSs are designed as collaborative modes that require exchanging information between the target and the server. Nevertheless, in some special scenes, such as illegal signal transmitter localization, emergency rescue, and anti-terrorism deployment, the targets’ accurate location information is crucial but more difficult to obtain when the sensors and target are not in a collaborative mode, which is usually denoted as passive localization. It is obvious that the passive localization system has more difficulty achieving stable and accurate positioning performance. Because of the requirement of target information, RSS fingerprinting-based methods and TOA-based methods are excluded for passive localization. TDOA methods are also not suitable because of their vulnerable measurements in the indoor scenario.

Fortunately, an effective DOA estimation scheme can achieve accurate target localization without synchronization or any other communication preparations. Additionally, DOA estimation can deal with multipath interference using antenna arrays. Therefore, the DOA estimation technique is a promising candidate for indoor passive localization applications.

DOA estimation is fundamental to many wireless sensing applications, especially RF source localization. Typical angle-of-arrival (AOA) estimation is performed using an antenna array with super-resolution algorithms, such as multiple signal classification (MUSIC) [16] and estimation of signal parameters via rotation invariance techniques (ESPRIT) [17].

Because of the complex wireless propagation in indoor environments, such as other systems, the indoor passive localization system must address challenges such as shadow fading, multipath propagation, and blockage. There are two major challenges for the DOA-based indoor passive localization systems. The received signals of the antenna array may be correlated resulting from a multipath effect that could invalidate the classic DOA estimation algorithm. Pre-processing, called spatial smoothing (SS), is needed to decorrelate the signals at the cost of losing the degree of freedom (DOF) [18,19]. Another problem is distinguishing the direct path from multiple candidates in the DOA spectra. In [10], a multipath suppression algorithm that leveraged changes in the wireless channel was proposed to identify the direct-path peak.

### 1.2. Contributions

In this paper, a novel indoor passive localization approach, denoted as the eigenspace-based DOA with direct-path recognition (ES-DPR) method is proposed. The signals are detected by the multiple omnidirectional antennas deployed in a uniform linear array (ULA) [20,21]. We utilize the eigenspace-based DOA (ES-DOA) algorithm, proposed in [22], to cope with the multipath signal in a novel style. The ES-DOA algorithm can distinguish one correlated signal without losing the DOF and estimate the signal strength value. And then, we develop a novel Direct-Path bearing Recognition (DPR) algorithm utilizing the estimated azimuth spectrum and RSS values from ES-DOA with ensemble learning methods, to identify the real bearing of the signal source. Hence, the proposed method is named as ES-DPR, which can be regarded as the combination of ES-DOA and DPR. Numerical simulations are conducted to verify the validity and superiority of the proposed method.

The novelty and advantages of the proposed ES-DPR method are summarized as follows:

1. Best to our knowledge, it is the first time for the indoor passive positioning method to combine the DOA and RSS estimate results of the array signals. ES-DPR shows good accuracy and robustness of DOA estimation in both uncorrelated and coherent cases. It shows significant superiority under low signal-to-noise ratios (SNR) and limited snapshot situations.
2. The proposed direct-path recognition algorithm can identify the true bearing of the target with only one base-station. It can achieve high direct-path recognition accuracy and distinguish the “no direct-path” case, which is common in the indoor environment. It is new and superior to the existing method.
3. Furthermore, the proposed method is easy for deployment. It can implement the localization job with single base station (one ULA) which is very attractive for practical applications.

### 1.3. Organization

The remainder of the paper is organized as follows: Section 2 introduces the preliminary knowledge and demonstrates the proposed ES-DPR method. In Section 3, we evaluate the performance of our approach through empirical experiments. Concluding remarks are presented in Section 4.

## 2. Preliminary and Methods

In this section, we first present the preliminaries of the proposed approach, including the signal model and the principle of the classic MUSIC algorithm, and we introduce the ES-DOA algorithm. Then, we demonstrate the direct-path recognition approach in detail.

### 2.1. Signal model

**Figure 1:**
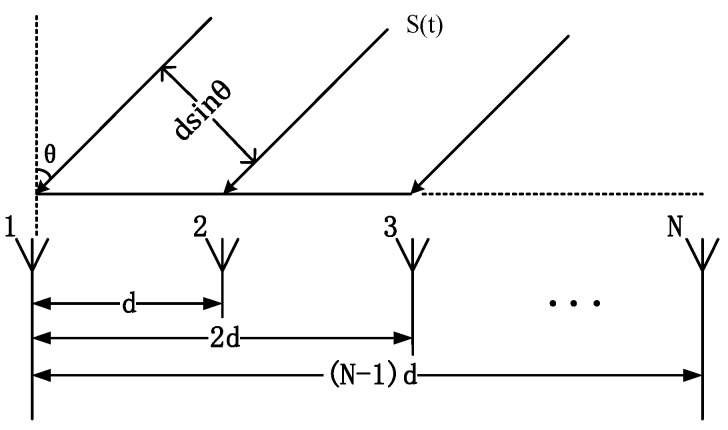
Diagram of a N-elements ULA.

Consider the array of *N* identical omnidirectional antennas uniformly spaced on a line composed of a ULA, depicted in Figure 1. The array steering vector of the ULA can be expressed in following form:

(1) $$a\left(\theta \right)={\left[1,e^{-j\frac{2\pi }{\lambda }dsin\theta },\ldots ,e^{-j\frac{2\pi }{\lambda }\left(N-1\right)dsin\theta }\right]}^{T}\;$$

where *θ* denotes the DOA of the source, *λ* is the wavelength of the incident signal. And *d* denotes the placement interval between each element of the array, which is usually set to *λ*/2 to obtain the maximum resolution [20].

Assume that there are *K* (*K* < *N*) sources with the source matrix *S* = [*s_1_*, *s_2_*, …, *s_K_*]***^T^***, and the receiving signals at the *N* elements ULA are given by:

(2) $$x={\left[x_{1},x_{2},\ldots ,x_{N}\right]}^{T}=AS+n=\overset{K}{\underset{i=1}{\sum }}a\left(\theta_{i}\right)s_{i}+n\;$$

where $A=\left[a\left(\theta_{1}\right),\;a\left(\theta_{2}\right),\ldots ,\;a\left(\theta_{K}\right)\right]$ is called the array steering matrix or manifold matrix of the source, *n* is white Gaussian noise with zero mean and *σ^2^* variance, *a*(*θ_i_*) is the normalized direction vector and *s_i_* is the transmitting signal of the *i*-th source.

The covariance matrix of the array signal ***R*** = *E*[*xx^H^*] is a key element for source detection. The eigen-decomposition is:

(3) $$\mathrm{EVD}\left(R\right)=U\Lambda U^{H}=\overset{N}{\underset{i=1}{\sum }}\lambda_{i}u_{i}u_{i}^{H}\;=U_{s}\Lambda_{s}U_{S}^{H}+U_{N}\Lambda_{N}U_{N}^{H}$$

where $\Lambda =\mathrm{diag}\left(\lambda_{1},\lambda_{2},\ldots ,\lambda_{N}\right)$ are the eigenvalues, which are sorted in non-decreasing order:

(4) $$\lambda_{1}\geq \lambda_{2}\ldots \geq \lambda_{K}\geq \lambda_{K+1}=\;\ldots \;=\lambda_{N}=\sigma^{2}\;$$

As shown by (3), the eigenvectors corresponding with the *K* larger eigenvalues construct the signal subspace *U_s_ =* [*u_1_*, *u_2_*, …, *u_K_*], and are the diagonal matrix *Λ_s_* composed of the *K* larger eigenvalues. The noise subspace is formed by a matrix containing the noise eigenvectors *U_N_ =* [*u_1+K_*, *u_2+K_*, …, *u_N_*] corresponding to the diagonal matrix *Λ_N_*.

### 2.2. MUSIC Algorithm and Spatial Smoothing Scheme

MUSIC is one of the most famous subspace-based algorithms for DOA estimation [16]. It is based on the observation that the steering vectors corresponding to signal components are orthogonal to the noise subspace eigenvectors:

(5) $$a^{H}\left(\theta_{i}\right)U_{N}U_{N}^{H}a\left(\theta_{i}\right)=0\;,\;i=1,2,\ldots ,K\;$$

and the spatial spectrum of *MUSIC* is then defined as:

(6) $$P_{MUSIC}\left(\theta \right)=\frac{1}{a^{H}\left(\theta_{i}\right){\hat{U}}_{N}{\hat{U}}_{N}^{H}a\left(\theta_{i}\right)}\;$$

where ${\hat{U}}_{N}$ denotes the noise eigenvectors of the observed signal.

The *MUSIC* spectrum is obtained by an exhaustive search over the impinging direction space, and the DOA estimations correspond to the *K* largest peaks in the *MUSIC* spectrum. Due to the multipath propagation effect in indoor environments, the signals may be highly correlated or coherent. In that case, the source covariance matrix is no longer nonsingular, and the rank of $U\Lambda U^{H}$ is lower than *K*. The MUSIC algorithm perceives the distinct incoming correlated signals as one superposed signal, resulting in false peaks in *P*(*θ*). To address this problem, the SS scheme is introduced to pre-treat the observed signals to recover the reduced-rank of the covariance matrix. The forward-backward spatial smoothing (FBSS) algorithm is the most commonly used method [19]. Its principle is to divide the original ULA into *L* overlapping uniform subarrays and introduce phase shifts between these subarrays. There are (*N* − *L* + 1) sensors in each subarray. After the FBSS is implemented, the covariance matrix is expressed as:

(7) $$R_{FB}=\frac{1}{2L}\;\overset{L}{\underset{i=1}{\sum }}F_{i}\left(R+JR^{*}J\right)F_{i}^{T}\;$$

(8) $$F_{i}=\left[0_{\left(N-L+1\right)\times \left(i-1\right)}\left|I_{\left(N-L+1\right)}\right|0_{\left(N-L+1\right)\times \left(L-i\right)}\right]\;$$

where *J* is the exchange matrix with ones on its anti-diagonal and zeros elsewhere.

However, compared to the uncorrelated case, the SS approaches realize decorrelation of the coherent signals at the cost of the DOF, which often leads to worse DOA estimations due to the reduction of the array aperture.

### 2.3. Eigenspace-Based DOA Algorithm ES-DOA

In [22], a novel DOA estimation algorithm named ES-DOA was proposed based on the investigation of MUSIC and improved MUSIC. The ES-DOA algorithm can achieve DOA estimation and signal source power estimation by making use of both the signal subspace and noise subspace characteristics.

The source covariance matrix can be reconstructed to a Toeplitz matrix as:

(9) $$R_{X}=R+I_{v}R^{*}I_{v}\;$$

where *I_v_* is the *N × N* exchange matrix with ones on its anti-diagonal and zeros elsewhere. The reconstructed matrix ***R_X_*** is decomposed, and the signal subspace part of ***R_X_*** is expressed as:

(10) $$R_{A}=U_{s}\Lambda_{s}U_{S}^{H}\;$$

The generalized inverse matrix of ***R_A_*** can be calculated as:

(11) $$R_{A}{}^{+}=U_{s}\Lambda_{s}{}^{-1}U_{S}^{H}\;$$

The frequency spectrum function in (6) can be modified as:

(12) $$P_{ES}\left(\theta \right)=\frac{a^{H}\left(\theta_{i}\right)R_{A}{}^{+}a\left(\theta_{i}\right)}{a^{H}\left(\theta_{i}\right)U_{N}U_{N}^{H}a\left(\theta_{i}\right)}\;$$

The power of the DOA is estimated by:

(13) $$P_{i}=\frac{1}{a^{H}\left(\theta_{i}\right)R_{A}{}^{+}a\left(\theta_{i}\right)}\;$$

The ES-DOA algorithm can achieve a high resolution and has strong anti-noise capability when dealing with coherent array signals. Its signal source power estimation ability is helpful for supplementing. These merits yield a significant advantage in indoor environments. Therefore, ES-DOA is a very suitable DOA estimation approach for indoor passive localization.

### 2.4. Direct path Recognition Approach

Due to the multipath propagation effect, the azimuth spectrum usually contains the direct-path peak and reflection-path peaks. It is a considerable challenge to distinguish the true direct-path bearing from the spectrum [23]. In [10], a multipath suppression method is motivated by the observation that the direct-path peak on the DOA spectrum is usually more stable than the reflection-path peaks when the transmitter, the receiver, or the objects between the two move a small distance. Since the reflection-path peaks are significantly sensitive to the propagation environment, the direct-path bearing can be identified by comparing the stability of the peaks on the DOA spectrum. Inspired by this observation, we propose a novel direct-path recognition approach that synthesizes the DOA and power estimation results of the target signal.

Assume that there are *K* (*K* < *N*) narrow-band far-field sources impinging on an *N* element ULA of antennas with the source matrix **s**(*t*) = [*s_1_*(*t*), *s_2_*(*t*), …, *s_K_*(*t*)]***^T^***. Assume we can obtain *M* groups of DOA and power estimation results during a phase of continuous time Δ*t*. Each group of results is calculated by the ES-DOA algorithm with *v* signal snapshots. The *M* groups of DOA and power estimation results are gathered into a joint set:

(14) $$\left[\Theta ,P\right]=\left[\begin{array}{cccc} \left(\theta_{1,1},p_{1,1}\right) & \left(\theta_{1,2},p_{1,2}\right) & \cdots & \left(\theta_{1,K},p_{1,K}\right)\\ \left(\theta_{2,1},p_{2,1}\right) & \left(\theta_{2,2},p_{2,2}\right) & \cdots & \left(\theta_{2,K},p_{2,K}\right)\\ \vdots & \vdots & \cdots & \vdots \\ \left(\theta_{M,1},p_{M,1}\right) & \left(\theta_{M,2},p_{M,2}\right) & \cdots & \left(\theta_{M,K},p_{M,K}\right) \end{array}\right]\;$$

The distribution of the DOA estimation *Θ* in (14) is analysed by using the histogram method [24,25]. Since the mode of the sample data can be calculated by the histogram method, it is beneficial for the analysis and processing of the azimuth estimation spectrum. The angle step Δ*ϕ* of the histogram is mainly determined by referring to the number of main multipath signals and the aperture of the ULA, and  Δϕ=5°~10° is suitable for most cases according to the simulations. The *K* bearing intervals with the most members that are supposed to correspond to the bearing of the K multipath signal, form the candidate bearing collection which is denoted as ***C*** = {*ϕ*_1_, *ϕ*_2_, …, *ϕ*_K_} based on the histogram method. Each category *ϕ* contains all the DOA and the corresponding power estimation results falling into the angle interval. The direct-path DOA estimation is supposed to be more stable and robust, the estimation results can be purified and some singular values and outliers resulting from the dynamic environment, are filtered out of the collection.

By calculating the 0-norm value $l^{0}$ from *θ* in each category *ϕ*, the mean value of the DOA and power $\left(\bar{\theta },\;\bar{p}\right)$ and standard deviation $\left(\sigma_{\theta },\;\sigma_{p}\right)$ of each category *ϕ* in set *C*, the statistic characteristic set of each category s(ϕ) is obtained and expressed as:

(15) $$s\left(\phi \right)=\left\{{\left(l^{0},\bar{\theta },\;\bar{p},{\;\sigma }_{\theta },\sigma_{p}\right)}_{1},\;{\left(l^{0},\bar{\theta },\;\bar{p},{\;\sigma }_{\theta },\sigma_{p}\right)}_{2},\;\cdots ,\;{\left(l^{0},\bar{\theta },\;\bar{p},{\;\sigma }_{\theta },\sigma_{p}\right)}_{K}\right\}\;$$

Several sub-classifiers can easily be obtained by comparing the values of the 0-norm, standard deviation of DOA and signal power estimation in s(ϕ). Then, we can construct a joint classifier *H*(*ϕ*’) by integrating the output results of each classifier:

(16) $$H=\{\begin{array}{c} h_{1}\left(\phi^{\prime }\right)=\underset{i}{\mathrm{argmax}}[{\left(l^{0}\right)}_{i}]\\ h_{2}\left(\phi^{\prime }\right)=\underset{i}{\arg min}\left[\;{\left(\sigma_{\theta }\right)}_{i}\right]\\ h_{3}\left(\phi^{\prime }\right)=\underset{i}{\mathrm{argmin}}\left[\;{\left(\sigma_{p}\right)}_{i}\right] \end{array}\;,\;i\in \left[1,K\right]\;$$

where the output of classifier *h_1_* is the index of one or several categories with the largest 0-norm value. As we consider that the direct-path signal can lead to a more stable and concentrated DOA estimate, a constraint $l_{j}^{0}\geq 0.5M$ is added to *h_1_* to ensure the rationality of the results. Apparently, the output result of classifier h_1_ would be *null* in some cases. The output result of classifier *h_2_* is the category index with the minimum standard deviation of DOA estimation, while *h_3_* is the category index with the minimum standard deviation of the signal power. In practice, the classify strategies of those sub-classifiers are supposed to be tunable to adapt to various indoor environments. For example, the output of *h_2_* can be the categories whose DOA standard deviations are smaller than a certain threshold value.

Due to the influence of interference and noise in the indoor environment, individual classifiers of *h_1_*, *h_2_* or *h_3_* are not robust and reliable enough. Thus, we utilize the joint classifier *H* to improve the robustness and anti-noise performance of the localization algorithm. Inspired by the idea of ensemble learning [24], the output of the joint classifier *H* is yielded from an absolute majority voting process [25]. The DOA estimation result $\hat{\theta }$ of the direct path is calculated by:

(17) $$\hat{\theta }=\{\begin{array}{l} {\bar{\theta }}_{j},\;\mathrm{if}\;\sum_{i=1}^{3}h_{i}^{j}>0.5\sum_{t=1}^{K}\sum_{i=1}^{3}h_{i}^{t}\\ “\text{No direct path}”,\;\mathrm{others}\; \end{array}\;$$

where *j* is the category index corresponding to the direct path from set *H*. And $h_{i}^{j}$ denotes the voting parameter, which is ‘1’ if the *j*-th category belongs to the outputs of classifier *h_i_*, otherwise the output is ‘null’.

If the *j*-th category obtains the most votes that exceed half of the total votes from the joint classifier *H*, then the mean value of DOA in category *j* is output as the bearing of the direct path of the signal source. Otherwise, it outputs “no direct path” in the case that there is no category owns the majority of the votes.

In ensemble learning, the commonly used combination strategies include the average method, the learning method and the voting method. The average method is the average processing of the output results of each weak classifier, which is obviously not suitable for direct-path discrimination. The learning method requires the combination of each classifier through the use of a learning machine with the sample training process and is not applicable to the scenario either.

In this paper, we choose the absolute majority vote as a portfolio strategy to deal with complex indoor positioning scenes. When the direct-path signal is very weak, for example, there may be no direct direction in the DOA estimation of the azimuth spectrum. While in a stationary no interference scenario, the multipath signal direction and the direction of the direct signal are stable, and the classifier can output multiple results. In this case, the absolute majority of votes may not be obtained by any category, and the output can be judged as “*no direct path*”, which can effectively improve the reliability of the system. The direct-path recognition process is illustrated in Algorithm 1.

**Algorithm 1** Direct Path Recognition (DPR)**Input:** *N:* number of the antennas; *K*: number of the multipath signals; *M*: number of the estimation groups; *v*: number of the signal snapshots; Δ*ϕ*: angel step; [Θ,P]: the DOA and power estimation joint set. $\left[\Theta ,P\right]=\left[\begin{array}{cccc} \left(\theta_{1,1},p_{1,1}\right) & \left(\theta_{1,2},p_{1,2}\right) & \cdots & \left(\theta_{1,K},p_{1,K}\right)\\ \left(\theta_{2,1},p_{2,1}\right) & \left(\theta_{2,2},p_{2,2}\right) & \cdots & \left(\theta_{2,K},p_{2,K}\right)\\ \vdots & \vdots & \cdots & \vdots \\ \left(\theta_{M,1},p_{M,1}\right) & \left(\theta_{M,2},p_{M,2}\right) & \cdots & \left(\theta_{M,K},p_{M,K}\right) \end{array}\right]$ **Output:** $\hat{\theta }$: the DOA estimation result of the direct path. **Process:** 1: Calculate the distribution of the DOA estimation *Θ* in (14) by using the histogram method. 2: Select the *K* intervals with the most members to form the candidate bearing collection ***C*** = {*ϕ*_1_, *ϕ*_2_, …, *ϕ*_K_} 3: Calculate the 0-norm value $l^{0}$, mean value of the DOA and power $\left(\bar{\theta },\;\bar{p}\right)$ and standard deviation $\left(\sigma_{\theta },\;\sigma_{p}\right)$ of each category *ϕ* in set *C.* 4: Obtain the statistic characteristic set of each category $s\left(\phi \right)=\left\{{\left(l^{0},\bar{\theta },\;\bar{p},{\;\sigma }_{\theta },\sigma_{p}\right)}_{1},\;{\left(l^{0},\bar{\theta },\;\bar{p},{\;\sigma }_{\theta },\sigma_{p}\right)}_{2},\;\cdots ,\;{\left(l^{0},\bar{\theta },\;\bar{p},{\;\sigma }_{\theta },\sigma_{p}\right)}_{K}\right\}$ 5: Calculate the index of one or several categories with the largest $l^{0}$ as the output of classifier *h*_1_ $h_{1}\left(\phi^{\prime }\right)=\underset{i}{\mathrm{argmax}}[{\left(l^{0}\right)}_{i}]$ 6: Calculate the category index with the minimum $\sigma_{\theta }$ as the output of classifier *h*_2_ $h_{2}\left(\phi^{\prime }\right)=\underset{i}{\arg min}\left[\;{\left(\sigma_{\theta }\right)}_{i}\right]$ 7: Calculate the category index with the minimum $\sigma_{p}$ as the output of classifier *h*_3_ $h_{3}\left(\phi^{\prime }\right)=\underset{i}{\mathrm{argmin}}\left[\;{\left(\sigma_{p}\right)}_{i}\right]$ 8: Calculate the DOA estimation result $\hat{\theta }$ of the direct path from the joint classifier *H* by using an absolute majority voting method $\hat{\theta }=\{\begin{array}{l} {\bar{\theta }}_{j},\;\mathrm{if}\;\sum_{i=1}^{3}h_{i}^{j}>0.5\sum_{t=1}^{K}\sum_{i=1}^{3}h_{i}^{t}\\ \text{no direct path},\;\mathrm{others}\; \end{array}\;$ 9: The direct-path recognition processing is done.

**Figure 2:**
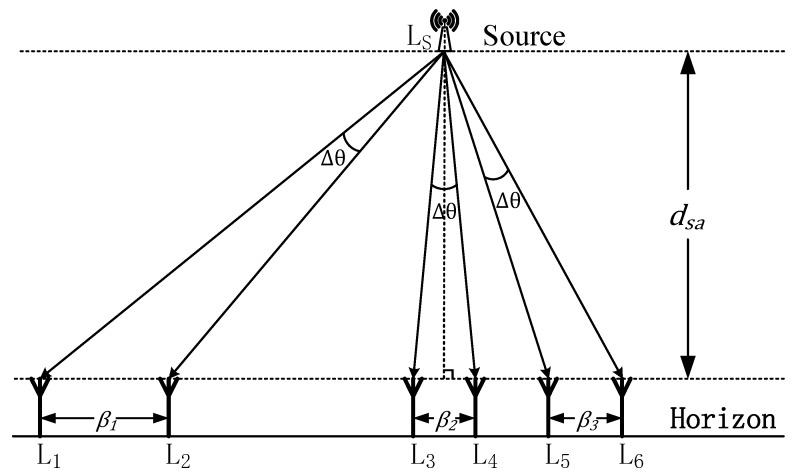
Diagram of the dynamic auxiliary decision mode.

If the direct path is not determined by the procedure mentioned above, then a dynamic auxiliary decision mode is started. As shown in Figure 2, the array moves slowly along the horizontal line of the ULA for a short distance during the measurement. L_s_ denotes the location of the signal source, L_i_ denotes the location of the array, and *β_j_* denotes the route distance of the *j*-th movement.

In order to alleviate the influence of the DOA shift Δθ resulting from the displacement of the array, the moving route is supposed to be within a small range. It can be observed from Figure 2. that, although the DOA shift angles are all Δθ when the array moves from L1 to L3, from L3 to L4, and from L5 to L6, the route distances are different. And the range value *β* is proportional to the absolute value of DOA. Hence, based on simple geometry, to ensure the DOA shift within Δθ, the maximum value of the displacement range *β* can be expressed as:

(18) $$\beta \leq 2d_{sa}\sin\left(\Delta \theta /2\right)$$

where *d_sa_* denotes the distance between the signal source and the horizontal line of the array, which is unknown in practice and assumed to be much longer than *β*. Fortunately, for the indoor scenario, the value of *d_sa_* can be estimated within a certain range referring to the layout of the building. For example, if *d_sa_* is set to be 10 m and Δθ is 1 degree, the maximum range value of *β* is 0.17 m calculated by (18).

In addition, the moving speed *V_ms_* should be slow enough that each group of the signal snapshots can be considered as being received at one spot:

(19) $$V_{ms}\leq \frac{F_{s}}{v}\delta d$$

where *F_s_* denotes the sampling rate of the snapshots, *v* is the number of the signal snapshots in one group, *d* is the placement interval of the array, and δ is a precision control factor belonging to (0,1). For instance, when the carrier frequency is 2.4 GHz, the sampling rate is 10 Mbps, *v* is 1000, *d* = λ/2 = 0.0625 m, and δ is 0.01, the moving speed *V_ms_* should be less than 6.25 m/s according to (19).

It should be noticed that the values of *β* and *V_ms_* have little impact on each other, and can be determined separately in practice. We repeat the DPR procedure during the moving course.

### 2.5. Crossover Localization

When the direct-path bearing is estimated by the procedure mentioned above, the target location can be conveniently determined by crossing the DOA results of two or more antenna arrays. When the target is static, it can also be located by one antenna array with multiple estimations in different locations [2].

## 3. Numerical Examples and Discussion

In this section, we employ numerical simulations to evaluate validity of the proposed method. The DOA estimation performance of the ES-DOA algorithm is evaluated first. And then we carry out the simulation of the proposed direct-path identification method.

### 3.1. Simulation Conditions

**Figure 3:**
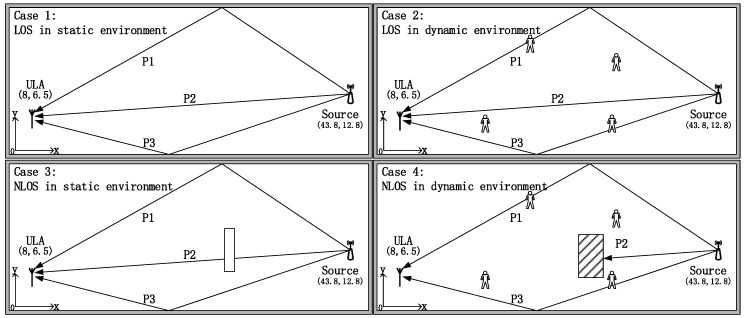
Diagram of different indoor environments.

To verify the universality and robustness of the proposed method, we simulate the performance of the ES-DOA algorithm combined with the proposed direct-path identification method in different conditions. To prevent the loss of generality, we divide the indoor environment into four different cases to contain more indoor environment situations. Set the positioning scene as (50 × 20) m indoor space, and the origin (0, 0) is at the bottom left. We assume that a single static target signal source is located at (43.8, 12.8), and a four elements ULA is located at (8, 6.5) of the area. As shown in Figure 3, the signal impinging on the ULA in three propagation paths P1, P2 and P3, and the bearings of these paths are −30°, −10° and 20°, respectively.

Furthermore, the received array signal is supposed to be the plane-wave incident narrow-band signal, and the spatial noise is zero mean, uniformly white complex Gaussian. The array antenna structure is an ideal ULA with four identical array elements. The spacing distance *d* is *λ*/2, and there is no phase offset between the receiving channels.

As shown in Figure 3, the first case simulates the situation that the signal source are in line-of-sight (LOS) in a static environment, while the second case in Figure 3 simulates the LOS signal source in a dynamic environment with moving people. The third case simulates the situation in which the direct-path of the source signal is attenuated by obstacles such as pillars or short walls in a static environment. The attenuation value arose from the wall is set to 12 dB, as it is reported that the signal absorption for brick walls is about 42 dB/m at 2.4 GHz [26]. The last case simulates the situation in which the direct-path of the source signal is completely blocked by the obstacles. The signal SNR condition is set to be 10 dB, and the number of signal snapshots is 100.

During the simulation, we utilize a simplified ray tracing method to calculate the bearing and power of the reflection-path, which is determined by the layout of the indoor scenario [26]. For each reflection-path signal, as we assumed, the walls are sufficiently smooth that specular reflections occur when the signals reach the boundaries. The reflection coefficient is assumed to be the same when the walls are homogenous.

The reflection coefficient can be calculated by:

(20) $$\Gamma_{⊥}=\frac{Z_{r}\cos\left(\psi_{i}\right)-\sqrt{1-{\left[Z_{r}\sin\left(\psi_{i}\right)\right]}^{2}}}{Z_{r}\cos\left(\psi_{i}\right)+\sqrt{1-{\left[Z_{r}\sin\left(\psi_{i}\right)\right]}^{2}}}\;$$

where *ψ_i_* denotes the grazing angle of the *i*-th propagation path, which is the complementary angle of the DOA of the *i*-th signal. *Z_r_* denotes the field impedance ratio between the cement wall and air, and can be calculated by

(21) $$Z_{r}=\frac{Z_{wall}}{Z_{air}}=\sqrt{\frac{\varepsilon_{\mathrm{air}}\mu_{\mathrm{wall}}}{\varepsilon_{\mathrm{wall}}\mu_{\mathrm{air}}}}\;$$

where *ε* denotes the relative permittivity, and *μ* denotes the relative permeability.

Assuming that there are only transverse electric reflections, the reflection power loss can be approximated by:

$$L_{r}\left(dB\right)=-10log10|\Gamma_{⊥}{|}^{2}$$

We suppose the relative permittivity and permeability of the cement walls are 1.5 and 1, while the relative permittivity and permeability of air both are 1. And the reflection coefficients and reflection power loss of P1 and P3 calculated by (20) and (21) are shown in Table 1.

The interference of the moving people are modeled as four reflectors at random spots within the area. The reflection power loss are set to 20 dB, and these reflectors would turn to the 6 dB attenuators when they are on the P1, P2, or P3 path. It should be noted that as the vertical height of the indoor scenario is much smaller than the scale of the horizontal direction, the signal’s azimuth can be considered DOA.

### 3.2. DOA Estimation Performance

In this section, the DOA estimation performance of the ES-DOA algorithm in indoor environment is evaluated based on the conditions of case 1 in Figure 3. The simulations are carried out with incoherent source signal and coherent source signal, respectively.

#### 3.2.1. Uncorrelated Source Signal Case

**Figure 4:**
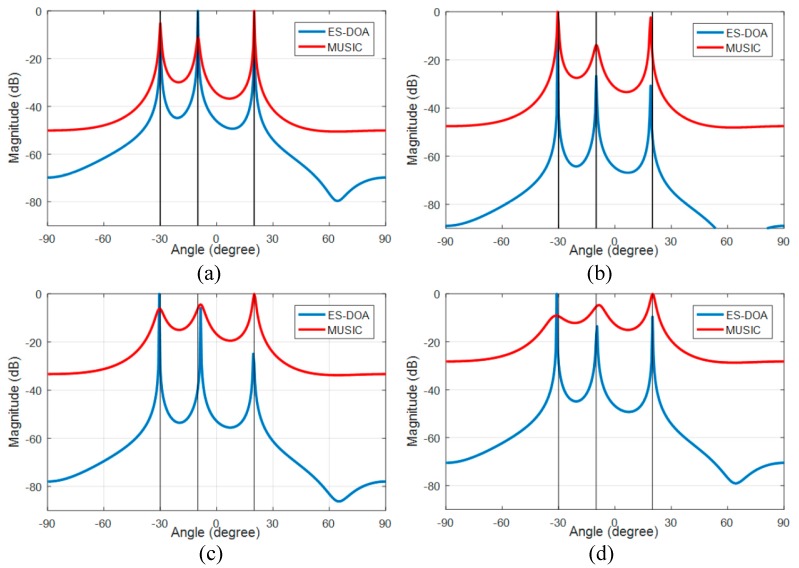
Azimuth spectrums of ES-DOA and MUSIC algorithms with 100 signal snapshots: (**a**) SNR = 20 dB; (**b**) SNR = 10 dB; (**c**) SNR = 5 dB; (**d**) SNR = 0 dB.

The performances of the ES-DOA algorithm under different SNR conditions in uncorrelated cases are compared with the classic MUSIC algorithm. The number of signal snapshots is set to 100. The DOA azimuth spectrum of the ES-DOA and MUSIC algorithm with incoherent received signals are shown in Figure 4, and the spatial noise is zero mean, uniformly white complex Gaussian, and the signal-to-noise ratio is 20 dB, 10 dB, 5 dB and 0 dB in case (a) to case (d), respectively. The true DOA values are indicated by the black vertical lines in the figures.

The peaks of the two spectra of the ES-DOA and the MUSIC algorithm are in good agreement with the true DOAs when the SNR conditions are good, as in case (a) and case (b). However, the spectra of the MUSIC algorithm significantly deteriorates with the decline in SNR, and the peaks become significantly blunt and inconspicuous when the SNR is low, as shown in case (c) and case (d). In contrast, the ES-DOA algorithm is robust to noise because the peaks in the spectra of the ES-DOA algorithm are still accurate and sharp when the SNR reaches 0 dB.

**Figure 5:**
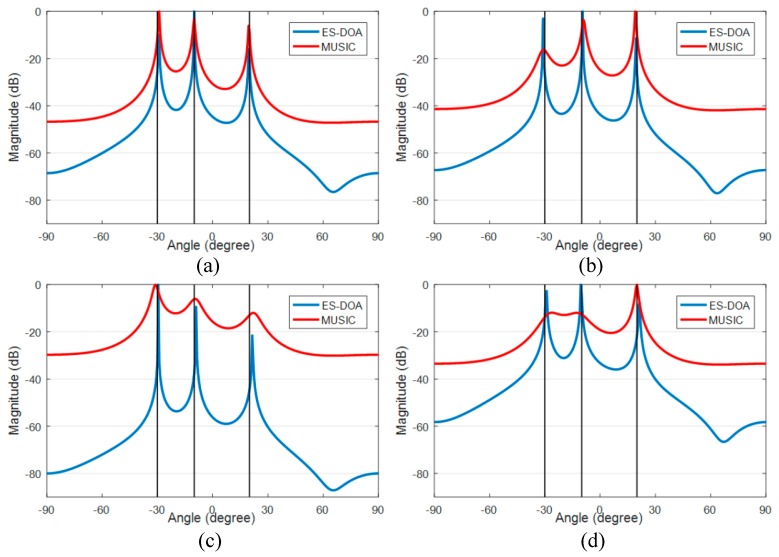
Azimuth spectrums of ES-DOA and MUSIC algorithms when SNR = 10 dB: (**a**) 1000 signal snapshots; (**b**) 200 signal snapshots; (**c**) 50 signal snapshots; (**d**) 10 signal snapshots.

Then we simulate the performance of the ES-DOA algorithm under different snapshot conditions in the uncorrelated case. The signal SNR condition is set to 10 dB. The DOA azimuth spectrum are shown in Figure 5, of which the number of snapshots is 1000, 200, 50 and 10 in case (a) to case (d), respectively. It can be observed that the ES-DOA algorithm can obtain accurate estimation results with limited received signal snapshot, while the performance of the classic MUSIC algorithm significantly relies on sufficient snapshots.

#### 3.2.2. Coherent Source Signal Case

**Figure 6:**
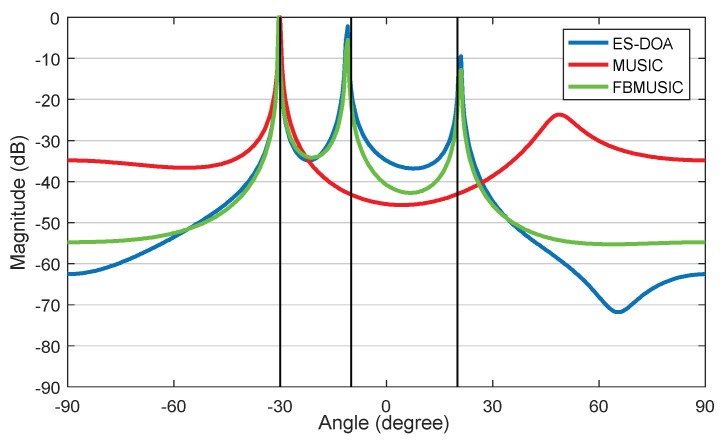
Azimuth spectrums of the various algorithms for coherent signals with 100 signal snapshots when SNR = 10 dB.

In the coherent source signal simulation, we assume that the signals from −10° and 20° are coherent. The SNR is 10 dB, and the snapshots number is 100. The performance of ES-DOA, FBSS-MUSIC and MUSIC dealing with the coherent signals is shown in Figure 6. The results indicate that ES-DOA and FBSS-MUSIC can both obtain accurate peaks while the classic MUSIC algorithm loses two peaks.

From the above simulations, it is fair to state that the ES-DOA algorithm is more robust than the classic MUSIC algorithm with incoherent signals and can achieve equivalent performance as the SS method when dealing with coherent source signals. In conclusion, the ES-DOA algorithm is a very attractive candidate for solving the DOA estimation problem in the indoor positioning scenario.

### 3.3. Direct-Path Identification Method

**Figure 7:**
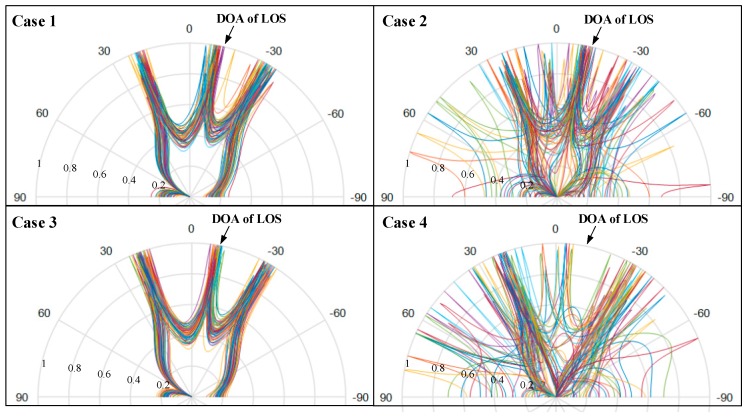
Azimuth spectrum synthesis of multiple continuous snapshot packets.

We simulate the performance of the ES-DOA algorithm combined with the proposed direct-path identification method in different conditions, as shown in Figure 3. The setup of the signal is same as that in Section 3.2.2. During each round of simulation, we combine 50 groups of DOA and power estimation results as the input of the ES-DOA algorithm. The integrated DOA estimation spectrum of each case is shown in Figure 7.

**Figure 8:**
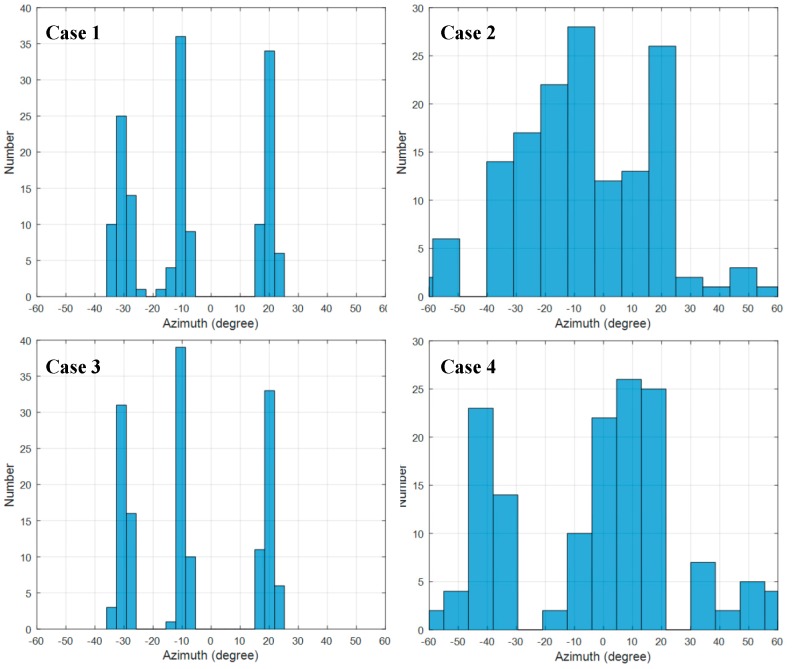
Statistical histogram of azimuth spectrum synthesis.

The corresponding distributions of the DOA estimation are demonstrated by the histogram in Figure 8. The angle step Δ*ϕ* of the histogram is set to 5°. From Figure 7 and Figure 8, it can be seen that the DOA estimation is stable and accurate when the indoor environment is static, as in cases 1 and 3, and the spectrum becomes severely messy when there are interferences in scenarios such as cases 2 and 4.

Based on the integrated spectrum and histogram data given above, Table 2 presents the outcome of the proposed direct-path recognition (DPR) algorithm of each case compared with the output of the multipath suppression method proposed in [15]. It is clear from the comparison results that the proposed DPR method can obtain accurate DOA estimation in cases 1, 2 and 3, and successfully identify the non-line-of-sight (NLOS) case, while the multipath suppression method fails to obtain the correct direction in cases 3 and 4.

To verify the advantage of our proposed method without losing generality, Monte Carlo simulations are carried out to measure the signal source direct-path recognition performance of the proposed DPR method and the multipath suppression method under different DOA incident conditions. We compared the output *θ_MS_* and *θ_DPR_* of the two methods by the direct-path recognition accuracy (dpRA), which is defined as:

(22) $$dpRA=\frac{\sum_{i=1}^{K}f\left(\left|{\hat{\theta }}_{i}-\theta_{i}\right|\right)}{K}\times 100\%,\;f\left(x\right)=\;\{\begin{array}{c} 1,\;x\leq \delta \\ 0,\;x>\delta \end{array}\;$$

where ${\hat{\theta }}_{i}$ and $\theta_{i}$ are the estimate and true value of the azimuth angle of the *i*-th Monte Carlo trial. K is the total number of Monte Carlo trials which is set to be 200 in our simulation. The estimated value is considered accurate as long as the deviation value $\left|{\hat{\theta }}_{i}-\theta_{i}\right|$ is smaller than the threshold value, which is set to 3° in the simulation.

**Figure 9:**
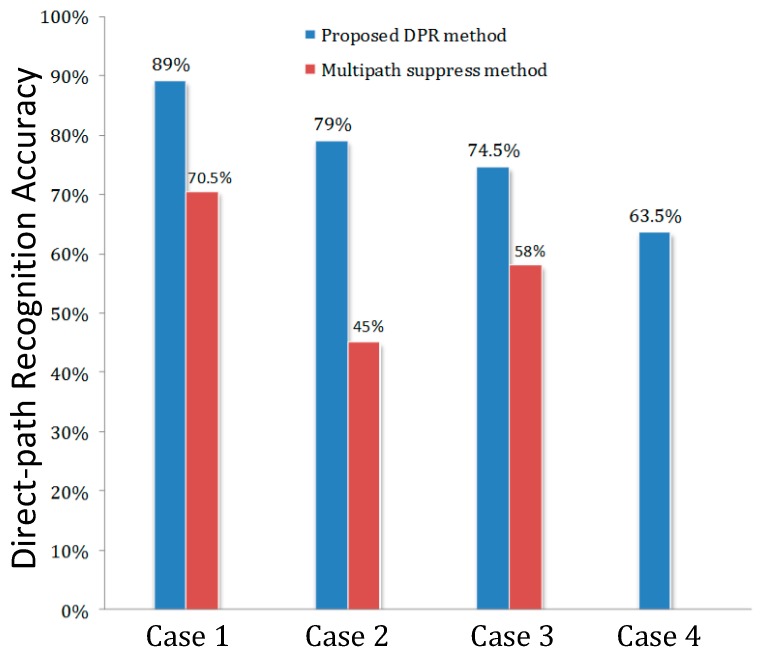
Direct-path recognition accuracy results from Monte Carlo simulations.

With 200 Monte Carlo trials, the results in Figure 9 clearly indicate that the proposed method offers a significantly higher dpRA over the multipath suppression method. In cases 1 and 3, the proposed DPR method improved the recognition accuracy by 18.5% and 15.5%, respectively, compared to that of the multipath suppression method. This advantage is much stronger when the scenario has considerable interference. As in case 2, the multipath suppression method shows a poor distinguishing rate which is below 50%. In contrast, the proposed DPR method improves remarkably in that the direct-path recognition accuracy is nearly 80%, which is even better than in case 3, due to the ensemble of DOA and power information. And in case 4, the proposed DPR method can distinguish the no direct-path condition by the chance of 63.5% while the traditional method fails under this situation.

## 4. Conclusions

In this paper, we have proposed a kind of DOA-based indoor passive localization method denoted as ES-DPR. We introduced the ES-DOA algorithm to deal with the coherent signals, and proposed a novel direct-path bearing recognition method to identify the real DOA of the signal source from multipath interference. Numerical simulations were conducted to verify the validity and superiority of the proposed method. The result shows that the ES-DOA algorithm can achieve high resolution and has strong anti-noise capability for dealing with coherent array signals, and the proposed DPR algorithm is reliable and robust in different indoor environments, even in the undetectable direct-path conditions.

The primary objective of this paper is to develop a passive localization method for a single static signal source, such as an illegal transmitter. Our next work will investigate how to locate and track a moving signal source with the ES-DRP algorithm. We initially assume that the DOA estimation algorithm is supposed to be enhanced with some algorithms, such as the extended Kalman filtering technique.

## Figures and Tables

**Table 1:** The grazing angles, reflection coefficients and reflection power loss of P1 and P3.

	P1	P3
$\psi_{i}$	60°	70°
$\Gamma_{⊥}$	0.27	0.39
$L_{r}$	11.4 dB	8.1 dB

**Table 2:** The outcome of the proposed DPR method and the multipath suppression method.

	DOA	${\hat{\theta }}_{MS}{\;}^{1}$	${\hat{\theta }}_{DPR}{\;}^{2}$
Case 1	−10°	−10.6°	−10.2°
Case 2	−10° (−12 dB)^3^	−11.2°	−10.7°
Case 3	−10°	19.6°	−10.4°
Case 4	NLOS	22.3°	No direct path

^1^${\hat{\theta }}_{MS}$ is the output of the multipath suppression method; ^2^ ${\hat{\theta }}_{DPR}$ is the output of the proposed DPR method; ^3^ Signal power of the direct-path is attenuated by 12 dB.

## References

[1] Gu Y., Lo A., Niemegeers I. (2009). A Survey of Indoor Positioning Systems for Wireless Personal Networks. IEEE Commun. Surv. Tutor..

[2] Yassin A., Nasser Y., Awad M., Ahmed A.-D., Ran L., Chau Y., Ronald R., Elias A. (2017). Recent Advances in Indoor Localization: A Survey on Theoretical Approaches and Applications. IEEE Commun. Surv. Tutor..

[3] Bahl P., Padmanabhan V.N. RADAR: An In-Building RF-Based User Location and Tracking System. Proceedings of the IEEE Infocom, Nineteenth Annual Joint Conference of the IEEE Computer and Communications Societies (Cat. No.00CH37064).

[4] Youssef M., Agrawala A. The Horus WLAN Location Determination System. Proceedings of the 3rd International Conference on Mobile Systems, Applications, and Services.

[5] Brunato M., Battiti R. (2005). Statistical learning theory for location fingerprinting in wireless LANs. Comput. Netw..

[6] He S., Chan S.H.G. (2017). Wi-Fi Fingerprint-Based Indoor Positioning: Recent Advances and Comparisons. IEEE Commun. Surv. Tutor..

[7] Chen Z., Wang J. (2018). GROF: Indoor Localization Using a Multiple-Bandwidth General Regression Neural Network and Outlier Filter. Sensors.

[8] Guvenc I. (2009). A survey on TOA based wireless localization and NLOS mitigation techniques. IEEE Commun. Surv. Tutor..

[9] Keunecke K., Scholl G. Deriving 2d Toa/Tdoa IEEE 802.11 g/n/ac Location Accuracy from an Experimentally Verified Fading Channel Model. Proceedings of the International Conference on Indoor Positioning and Indoor Navigation.

[10] Vasishty D., Kumar S., Katabi D. Decimeter-Level Localization with a Single WiFi Access Point. Proceedings of the USENIX Symposium on Networked Systems Design and Implementation.

[11] Yang C., Shao H.R. (2015). WiFi-based indoor positioning. Commun. Mag. IEEE.

[12] Exel R., Gaderer G., Loschmidt P. (2010). Localisation of Wireless LAN Nodes Using Accurate TDoA Measurements. Proceedings of the Wireless Communications & Networking Conference.

[13] Xie T., Zhang C., Li Y., Jiang H., Wang Z. (2017). An Enhanced TDoA Approach Handling Multipath Interference in Wi-Fi Based Indoor Localization Systems. Proceedings of the 60th International Midwest Symposium on Circuits and Systems.

[14] Kotaru M., Joshi K., Bharadia D., Katti S. SpotFi: Decimeter Level Localization Using WiFi. Proceedings of the ACM.

[15] Xiong J., Jamieson K. ArrayTrack: A Fine-Grained Indoor Location System. Proceedings of the USENIX Symposium on Networked Systems Design and Implementation.

[16] Schmidt R., Schmidt R.O. (1986). Multiple Emitter Location and Signal Parameters Estimation. IEEE Trans. Antennas Propag..

[17] Roy R., Kailath T. (2002). ESPRIT-estimation of signal parameters via rotational invariance techniques. IEEE Trans. Acoust. Speech Signal Proc..

[18] Shan T.J., Wax M., Kailath T. (1985). On spatial smoothing for direction-of-arrival estimation of coherent signals. IEEE Trans. Acoust. Speech Signal Proc..

[19] Pillai S.U., Kwon B.H. (1989). Forward/backward spatial smoothing techniques for coherent signal identification. IEEE Trans. Acoust. Speech Signal Proc..

[20] Trees H.L.V. (2002). Optimum Array Processing: Part IV of Detection, Estimation and Modulation Theory.

[21] Haykin S., Justice J.H., Owsley N.L., Yen J.L., Kak A.C. (1997). Array Signal Processing. Proceedings of the IEEE Signal Processing Workshop on Higher-order Statistics.

[22] Zhang X., Lv W., Shi Y., Zhao R., Xu D. (2007). A Novel DOA Estimation Algorithm Based on Eigen Space. Proceedings of the International Symposium on Microwave.

[23] Yu K., Guo Y.J. (2009). Statistical NLOS Identification Based on AOA, TOA, and Signal Strength. IEEE Trans. Veh. Technol..

[24] Hastie T., Tibshirani R., Friedman J. (2009). Ensemble Learning. The Elements of Statistical Learning.

[25] Bishop C.M. (2006). Pattern Recognition and Machine Learning (Information Science and Statistics).

[26] Stijn W., Lieven S. (2017). Indoor Multipath Assisted Angle of Arrival Localization. Sensors.

